# Characterization and Bio-Typing of Multidrug Resistance Plasmids From Uropathogenic *Escherichia coli* Isolated From Clinical Setting

**DOI:** 10.3389/fmicb.2019.02913

**Published:** 2019-12-18

**Authors:** Sandip Kumar Mukherjee, Mandira Mukherjee

**Affiliations:** Department of Biochemistry and Medical Biotechnology, Calcutta School of Tropical Medicine, Kolkata, India

**Keywords:** uropathogenic *E. coli*, MDR, PBRT, co-transmission, T4SS, pili

## Abstract

Urinary tract infection is primarily caused by *Escherichia coli*. Multidrug resistance and their rapid dissemination in this pathogenic microbe complicate therapeutic strategies and threaten public health. Conjugation systems responsible for interbacterial transmission of antibiotic resistance are plasmid-encoded and can be classified as the P, F, and I types. Specific pili types and pili associated proteins were related to the transfer among this gram-negative organism and were thought to depend on contacts created by these structures at the time of DNA transport. In this study, conjugation system types of the plasmids that harbor multidrug resistant genes (*aac-1b-cr*, *oqxAB*, *qnrB*, *qnrS*, *bla*_TEM_, *bla*_OXA_) amongst 19 *E. coli* uropathogenic isolates were characterized under ciprofloxacin/ceftazidime selection individually by pili and pili associated gene types. Investigations indicated incidence of single plasmid of multiple replicon type amongst the transconjugants. *bla*_TEM_, *bla*_CTX–M_, *bla*_OXA_, *aac-1b-cr*, *oqxAB*, *qnrB*, *qnrS* genes in varied combination were observed to be successfully co-transmitted against ceftazidme/ciprofloxacin selection. Seven primer pair sets were selected that encodes pili and pili associated genes (*traF*, *trwJ*, *traE*, *trhE*, *traG*, *pilM*, *pilx4*) by nucleotide database search tools using annotated plasmids of different incompatibility types to assign the conjugation system type of the transmissible resistant plasmids by PCR. *tra*F was predominant irrespective of drug selection that indicated F-type conjugation system was responsible for transmission of resistant plasmids which results in the rapid dissemination of antibiotic resistance in the isolates screened. Therefore this is a first report of its kind that investigated pili and pili associated genes to bio-type multidrug resistant plasmids and their transmission in clinical settings amongst uropathogenic *E. coli* circulated in the eastern part of India.

## Introduction

Urinary tract infection (UTI) is known to cause significant levels of morbidity and mortality in developed countries and has become a public health concern. *Escherichia coli* is reported to be one of the primary etiologic agents that cause UTI. Emergence of antibiotic resistance in this bacterial pathogen is recognized as one of the greatest threats to global healthcare management system (reviewed in Hadifar et al., 2017).

Multidrug resistant (MDR) isolates causing UTI have serious implications for the empiric therapy against pathogenic isolates and for the possible co-selection of antimicrobial resistant pathogens. There are several factors responsible for dissemination of antimicrobial resistance genes among these pathogens, and plasmid-mediated transfer has been considered one of the most important mechanisms for the horizontal transfer of multidrug resistance (Akingbade et al., 2014). The most widely used plasmid classification scheme is PCR based replicon typing (PBRT) which exploited loci encoding plasmid replication machinery (Carattoli et al., 2005, 2014) which provided insights into resistance plasmid epidemiology, such as whether resistance dissemination involves diverse plasmids or one dominant “epidemic” type (Valverde et al., 2009).

A recent review summarized the major plasmid families that are currently emerging in MDR *Enterobacteriaceae* strains isolated in several parts of the world including those conferring resistance to important antibiotics such as extended-spectrum cephalosporins, fluoroquinolones, and aminoglycosides (Carattoli, 2009). Certain replicon types were found to be associated with MDR as well as with bacterial disease outbreaks (Huang et al., 2012). In turn, this can be useful for epidemiologic surveillance and the development of strategies to prevent their spread (Huang et al., 2012; Dehbanipour et al., 2016). However, evidence of multiple replicon types among individual resistant plasmid questioned the fidelity of the classification technique and demands further characterization of the resistant plasmids to address strategies to prevent their spread.

Conjugation systems play key role for interbacterial transfer of antibiotic resistance genes, pathogenicity, and genes encoding other traits of potential benefit to the bacterial host. The plasmid encoded type IV secretion systems (T4SSs) that belongs to IncP, IncF, and IncI identified in *E. coli* and other species of *Enterobacteriaceae* function exclusively in conjugative DNA transfer and had established its role in organizing bacterial genomes and transmission of antibiotic resistance in clinical settings (Christie, 2016). In IncI plasmid systems, which have both, type IV pili (T4P) and conjugative pili, the former involved in binding of the donor to the recipient cells and after binding, the F-pilus retracts and a stable association between donor and recipient cells initiated by a process of mating pair stabilization (Mps) that involves the translocation of a structure containing TraG into the recipient cell periplasm which further signals the commencement of conjugative DNA transport and replication by the donor cell (Hazes and Frost, 2008). Therefore specific pili types and pili associated proteins were related to the transfer of IncP, IncF and IncI plasmids among this gram-negative organism which was thought to depend on contacts created by these structures at the time of DNA transport (Christie, 2016).

In this study, we characterized multidrug resistant plasmids obtained from uropathogenic *E. coli* isolates collected from hospitalized patients with respect to β-lactamase and quinolone resistant gene acquisition and their co-transmission by conjugation, a common phenomenon in the natural habitat. Moreover the type of conjugation system followed by the clinical plasmids was explored based on pili and pili associated gene types to understand and evaluate potential of horizontal gene transfer among these gram negative pathogen.

## Materials and Methods

### Bacterial Culture

A total of 80 urine samples were collected from Carmichael Hospital for Tropical Diseases, Kolkata from patients suffering from UTI. *E. coli* were detected in the urine culture positive samples by standard biochemical tests and cultured in Luria Bertani Broth (Hi-Media Laboratories, India). The study protocol was approved by the institutional ethical committee.

### Antibiotic Susceptibility Testing

*Escherichia coli* isolates were tested by Kirby Bauer disk diffusion method on Muller Hinton agar plates using following antibiotic disks; amikacin (AK; 10 μg), ceftazidime (CAZ; 30 μg), cefotaxime (CTX; 30 μg), cefoxitin (CX; 30 μg), ciprofloxacin (CIP; 5 μg), levofloxacin (LE; 5 μg), cotrimoxazole (COT; 25 μg), nitrofurantoin (NIT; 300 μg), and imipenem (IPM; 10 μg) (Hi-Media laboratories, India). *E. coli* ATCC 25922 was used as quality control strain. Isolates resistant, intermediate resistant and sensitive to individual antibiotics were determined by the zone of inhibition that was interpreted following CLSI (2016) guidelines (CLSI, 2016). An isolate was considered as multidrug resistant if it was resistant to ≥3 classes of antibiotics. Extended spectrum β-lactamase production was determined in isolates that were resistant to cephalosporin (Sharma et al., 2013).

### Plasmid Isolation, Bacterial Conjugation, and PCR Analysis

Plasmid DNA was prepared by alkaline lysis method and electophoresed on 0.8% agarose gels and visualized by Gel documentation system (BioRad) (Khadgi et al., 2013). Plasmid bands of varied size (approximate) were detected using molecular weight marker, lambda/*Hin*dIII double digest. Conjugal transfer of plasmid to *E. coli* J53AzideR recipient strain was performed by broth mating assay (Ghosh and Mukherjee, 2016). Transconjugants were screened by double selection method on MacConkey agar plates containing sodium azide (100 μg/ml) and ceftazidime (30 μg/ml), or ciprofloxacin (5 μg/ml), respectively. Plasmids from the clinical isolates and the transconjugants were screened by PCR for the detection of β-lactamase genes; *bla*_TEM_, *bla*_CTX–M_, *bla*_OXA_ (Tayebi et al., 2016; Bandyopadhyay and Mukherjee, 2017) and PMQR genes; *oqxAB, qnrA, qnrB, aac(6′)-Ib-cr, qepA* genes with gene specific primers (Tayebi et al., 2016). Purity of the plasmid preparations isolated from transconjugants was ascertained by PCR with chromosomal gene (*fimH*, *papC*, *cnf1*) specific primers (Supplementary Table S1) and RFLP analysis. Discrete difference in the RFLP pattern was observed amongst the plasmid DNA which was absent in genomic DNA isolated from the clinical counterpart that was used as a control (Supplementary Figures S1, S2, S3).

### Plasmid Replicon Typing

Incompatibility groups of the plasmids isolated from the transconjugants were determined by PCR-based replicon typing (PBRT) method using IncFrep, F1B, N, I1, A/C, H1, X, Y, L/M, W by specific PCRs as described previously (Carattoli et al., 2005).

### *In silico* Analysis

An *in silico* analysis was carried out using GenBank BLAST^1^ on *E. coli* annotated plasmids of IncF, IncI1, IncN, IncW, IncHI, IncA/C, IncX replicon types. However, due to insufficient data on the various incompatibility types from *E. coli*, the *in silico* analysis was extended to some plasmids of other *Enterobactereace* family. For the seven Inc groups of plasmids incident in this study pili and pili associated genes involved in conjugation event was used as template for nucleotide-Search^2^ to identify candidate genes which were specific for individual incompatibility types (Bousquet et al., 2015). The target genes were further validated by blastN (see footnote 2) and ClustalW2 software^3^. Primer pairs covering most sequences in each family were designed using FastPCR^4^ (Table 1 and Supplementary Figure S4).

**TABLE 1:** Primers used in this study.

**Annotated plasmids**	**Primer name**	**Sequence (5′-3′)**	**Length (bp)**	**Amplicon size (bp)**
R100	*traF*-fp	CCGGCTGCAGAATTACTGGAC	21	114
	*traF*-rp	CTGCAGTACAGCCATTACAACGG	23	
R64	*pilM*-fp	ATGGGATGGCTGGTCATGGCC	21	232
	*pilM*-rp	CCCTGTCACTCCGGACTCCC	20	
R46	*traE*-fp	GCTAATAAAAAAACAGGGC	18	624
	*traE*-rp	AACCCGGAAGTTAACTGA	18	
R388	*trwJ*-fp	TGAAGAAGCTGGTTATGAC	19	560
	*trwJ*-rp	TATCCAGAACGAAACGAC	18	
R27	*trhE*-fp	ATGAAACTTCTCAGCAGGT	19	442
	*trhE*-rp	AACATCGGTTTTGTTACAAC	20	
RA1	*traG*-fp	TTGTTGTTGATGCTCTAAACA	21	350
	*traG*-rp	ATTGCTTTTATTATCGTGATG	21	
R6K	*pilx4*-fp	CGAGTTTTCTCAGATCAGC	19	358
	*pilx4*-rp	CTAAATCCCTCCTTGCTG	18	

## Results

### Bacterial Isolates

In the present study 50 urine samples yielded significant growth out of 80 samples collected from patients suffering from UTI. 19 *E. coli* isolates were identified from the 50 urine culture positive samples by routine biochemical analysis.

### Antibiotic Resistance

All 19 *E. coli* isolates were resistant toward ciprofloxacin, ceftazidime, cefotaxime, cefipime, amikacin, levofloxacin, nitrofurantoin, and imepenem except 2, 3, 4, 8, and 8 out of the 19 which showed intermediate resistance against amikacin, levofloxacin, nitrofurantoin and imepenem, respectively.

### Acquired Plasmids and Resistant Gene Profiles

**FIGURE 1:**
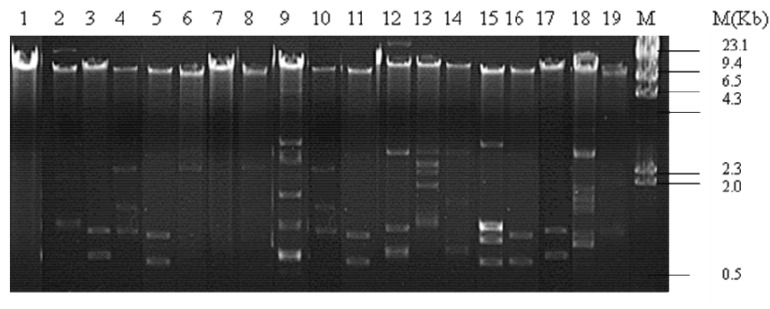
Plasmid pattern of clinical uropathogenic *E. coli* isolates. Lanes 1–19, plasmid extracted from the clinical isolates (*n* = 19) and electrophoresed on 0.8% agarose gel. M, lambda/*Hin*dII DNA ladder.

Plasmid profiling of all 19 isolates showed presence of one to seven plasmids of approximate sizes ranging from ∼12 to ∼1 kb. Prevalence of a plasmid of an approximate size in the range of ∼12 kb was common in all isolates. 8 out of 19 isolates exhibited three plasmid bands while one, two, four and five/seven plasmid bands were detected in 2/19, 1/19, 4/19, and 4/19 of the isolates, respectively (Figure 1). Plasmids from all 19 isolates indicated presence of β-lactamase genes *bla*_TEM_, *bla*_CTX–M_, *bla*_OXA_ and the PMQR genes *aac(6’)1b-cr*, *oqxAB*, *qnrB* in varied combinations. *bla*_TEM_ and *bla*_OXA_ was observed in all 19 isolates in combination with *bla*_CTX–M__–__15_ type (13/19), and the PMQR genes; *aac(6′)1b-cr* (14/19), *oqxAB*(2/19), *qnrB*(7/19), and *qnrS* (1/19), respectively (Table 2). Therefore it may be assumed that *bla*_TEM_ and *bla*_OXA_ genes harbored by the clinical isolates donot encode ESBLs and exhibited β-lactam- β-lactamase inhibitor resistant phenotype (data not shown).

**TABLE 2:** Resistance genes in the clinical uropathogenic *E. coli* isolates (*n* = 19).

**Clinical isolates**	**Resistance genes**						
	**β-lactamase**			**PMQR**			
	***bla*_TEM_**	***bla*_CTX–M_**	***bla*_OXA_**	***aac-1b-cr***	***oqxAB***	***qnrB***	***qnrS***
EC175	+	+	+	+	+	−	−
EC 176	+	+	+	+	−	−	−
EC 177	+	−	+	+	−	−	−
EC 178	+	−	+	+	−	+	−
EC 180	+	+	+	−	−	−	+
EC183	+	+	+	+	−	−	−
EC 185	+	+	+	+	−	+	−
EC 187	+	−	+	+	−	−	−
EC 194	+	−	+	+	−	−	−
EC 195	+	+	+	+	−	−	−
EC 197	+	+	+	+	−	−	−
EC 207	+	+	+	+	+	+	−
EC 211	+	+	+	−	−	+	−
EC 215	+	+	+	−	−	+	−
EC 216	+	−	+	−	−	+	−
EC 217	+	+	+	−	−	+	−
EC 219	+	+	+	+	−	−	−
EC 222	+	−	+	+	−	−	−
EC 224	+	+	+	+	−	−	−

### Conjugal Transfer

Transfer of resistance determinants from all 19 clinical *E. coli* donors to the *E. coli* J53AziR recipients strain was observed against β-lactam antibiotics (ceftriazone, ceftazidime, cefotaxime) and fluoroquinolones (ciprofloxacin, levofloxacin). The β-lactamase genes *bla*_TEM_, *bla*_CTX–M_, *bla*_OXA_ and the PMQR genes *aac(6′)1b-cr,oqxAB, qnrB*, respectively were transferred in various combinations (Table 3).

**TABLE 3:** Plasmid replicon types and resistance gene distribution among the transconjugants.

**Drug selection**	**Transconjugants**	**β-lactamase genes**			**Replicon(s)**	**PMQR genes**			
		***bla*_TEM_**	***bla*_CTX–M_**	***bla*_OXA_**		***aac-1b-cr***	***oqxAB***	***qnrB***	***qnrS***
C	TCa175	+	+	−	Frep, I1	+	+	−	−
E	TCa176	+	+	+	Frep	−	−	−	−
F	TCa177	+	−	+	Frep, I1	+	−	−	−
T	TCa178	+	−	+	F1B	−	−	−	−
A	TCa180	+	+	+	F1B	−	−	−	−
Z	TCa183	+	+	+	Frep,F1B	+	−	−	−
I	TCa185	+	+	−	Frep, I1	−	−	−	−
D	TCa187	+	−	+	Frep	−	−	−	−
I	TCa194	+	−	+	Frep,F1B	−	−	−	−
M	TCa195	+	+	+	Frep,F1B	+	−	−	−
E	TCa197	+	+	+	F1B	+	−	−	−
	TCa207	+	+	+	Frep	+	+	−	−
	TCa211	+	+	−	Frep	+	−	−	−
	TCa215	+	+	−	Frep, I1	+	−	−	−
	TCa216	+	−	+	Frep, I1	−	−	−	−
	TCa217	+	+	+	F1B	−	−	−	−
	TCa219	+	+	+	F1B	−	−	−	−
	TCa222	+	−	+	F1B	−	−	−	−
	TCa224	+	+	+	Frep,N	+	−	−	−
C	TCi175	+	−	−	Frep,W,X	+	−	−	−
I	TCi176	+	+	+	Frep,A/C,F1B,W	+	−	−	−
P	TCi177	+	−	+	Frep,W	+	−	−	−
R	TCi178	+	−	−	Frep,I1,L/M,W	+	−	−	−
O	TCi180	+	+	−	Frep,A/C,F1B,Y,W,X	−	−	−	+
F	TCi183	−	+	+	Frep,F1B,W	+	−	−	−
L	TCi185	+	−	−	Frep	+	−	+	−
O	TCi187	+	−	−	Frep,I1	+	−	−	−
X	TCi194	+	−	+	Frep,F1B	+	−	−	−
A	TCi195	+	−	+	Frep	+	−	−	−
C	TCi197	+	+	+	Frep	+	−	−	−
I	TCi207	+	−	−	Frep,FIB,W	+	−	+	−
N	TCi211	+	+	+	Frep,A/C,F1B,W	−	−	+	−
	TCi215	+	−	+	Frep,F1B,W	−	−	+	−
	TCi216	+	−	+	Frep,L/M,W,X	−	−	+	−
	TCi217		+	+	Frep	−	−	+	−
	TCi219	+	−	+	Frep,F1B,W,X	+	−	−	−
	TCi222	+	−	−	Frep,A/C,F1B, F11S,H1,W,X	+	−	−	−
	TCi224	+	−	+	I1,W	+	−	−	−

Selection of transconjugants against ceftazidime and ciprofloxacin independently showed acquisition of resistant plasmids with both single as well as multiple replicon types with universal presence of IncF type plasmid. Single replicon type plasmid which belonged to IncF type (IncFrep) was observed in four transconjugants selected against ciprofloxacin and 10 (IncFIB; 6, IncFrep; 4) selected against ceftazidime respectively. Additionally presence of multiple replicon Inc type plasmids (I1, W, X, Y, A/C, L/M, HI) were detected in transconjugants obtained under ciprofloxacin compared to IncF and IncI1 obtained under ceftazidime selection (Table 3).

### Identification of Pili and Pili Associated Genes

Genes encoding pili and pili associated proteins were members of the bacterial T4SS system and actively participates facilitating the IncF and IncP bacterial conjugation machinery. However, pili associated with IncI conjugation system enhances the process of conjugation without directly playing a role in the transfer mechanism. Nucleotide sequences of genes encoding pili and pili associated proteins were identified in the annotated plasmids R100 (Ac No. NC_002134.1), R64 (Ac.No. AP005147.1.), R46 (Ac.No NC_003292.1.), R388 (Ac.No. NC_028464.1), R27 (Ac.No NC_002305.1), pRA1 (Ac.No NC_012885.1), R6K (Ac No AJ006342.1) assigned to IncF, IncI, IncN, IncW IncH, IncA/C, and IncX plasmid incompatibility types, respectively (Table 4) to characterize the conjugation system type in the transconjugants that harbor plasmid of multiple replicon types.

**TABLE 4:** Pili and pili associated genes in annotated plasmids assigned to specific plasmid replicon types.

**Replicon**	**Annotated**	**Accession No.**	**Genes encoding**	
	**plasmids**			
			**Pili**	**Pili associated proteins**
IncF	R100	NC_002134.1		*traF*
IncI1	R64	AP005147.1	*pilM*	
IncN	R46	NC_003292.1		*traE*
IncW	R388	NC_028464.1		*trwJ*
IncH	R27	NC_002305.1		*trhE*
IncA/C	pRA1	NC_012885.1		*traG*
IncX	R6K	AJ006342.1	*pilx4*	

Plasmid isolated from the 16 and 3 transconjugants obtained under ceftazidime selection showed presence of *tra*F and *pil*M, respectively (Table 5). Overall similar results were observed in transconjugants selected under ciprofloxacin pressure, however, the transconjugants that exhibited presence of *pil*M amongst the later group were *tra*F positive amongst the former respectively (Table 5). Moreover combination of *tra*F and *pil*M or other pili associated genes *trw*J, *tra*E, *trh*E, *tra*E, and *pil*x4 were not detected. Presence of *tra*F and *pil*M amongst the transconjugants were further confirmed by southern blot hybridization using the *traF* and *pilM* cloned amplicons as probes (data not shown).

**TABLE 5:** Conjugal plasmid types associated with transmissible resistant plasmids in transconjugants.

**Drug selection**	**Transconjugants**	**Pili and pili associated genes**							**Conjugal plasmid types**
		***traF***	***pilM***	***traE***	***trwJ***	***trhE***	***traG***	***pilx4***	
C	TCa175	+	−	−	−	−	−	−	F
E	TCa176	+	−	−	−	−	−	−	F
F	TCa177	−	+	−	−	−	−	−	I
T	TCa178	+	−	−	−	−	−	−	F
A	TCa180	+	−	−	−	−	−	−	F
Z	TCa183	+	−	−	−	−	−	−	F
I	TCa185	+	−	−	−	−	−	−	F
D	TCa187	+	−	−	−	−	−	−	F
I	TCa194	+	−	−	−	−	−	−	F
M	TCa195	+	−	−	−	−	−	−	F
E	TCa197	+	−	−	−	−	−	−	F
	TCa207	+	−	−	−	−	−	−	F
	TCa211	+	−	−	−	−	−	−	F
	TCa215	−	+	−	−	−	−	−	I
	TCa216	−	+	−	−	−	−	−	I
	TCa217	+	−	−	−	−	−	−	F
	TCa219	+	−	−	−	−	−	−	F
	TCa222	+	−	−	−	−	−	−	F
	TCa224	+	−	−	−	−	−	−	F
C	TCi175	+	−	−	−	−	−	−	F
I	TCi 76	+	−	−	−	−	−	−	F
P	TCi177	+	−	−	−	−	−	−	F
R	TCi178	−	+	−	−	−	−	−	I
O	TCi 80	+	−	−	−	−	−	−	F
F	TCi183	+	−	−	−	−	−	−	F
L	TCi185	+	−	−	−	−	−	−	F
O	TCi187	−	+	−	−	−	−	−	I
X	TCi194	+	−	−	−	−	−	−	F
A	TCi195	+	−	−	−	−	−	−	F
C	TCi197	+	−	−	−	−	−	−	F
I	TCi207	+	−	−	−	−	−	−	F
N	TCi211	+	−	−	−	−	−	−	F
	TC i215	+	−	−	−	−	−	−	F
	TCi 216	+	−	−	−	−	−	−	F
	TCi217	+	−	−	−	−	−	−	F
	TCi219	+	−	−	−	−	−	−	F
	TCi222	+	−	−	−	−	−	−	F
	TCi224	−	+	−	−	−	−	−	I

## Discussion

In this study, we focused on the genetic classification of the transmissible plasmids isolated from multidrug resistant uropathogenic *E. coli* which has an impact on dissemination of drug resistance amongst this uropathogen. Multidrug resistance is an emerging threat propagated in this pathogenic microbe. Worldwide and nationwide studies showed different resistance rates against several antibiotic groups (reviewed in Hadifar et al., 2017). Moreover recent reports from India indicated an alarming rise in the incidence of multidrug resistance (i.e., simultaneous resistance to various class of antibiotics, such as aminoglycosides, cephalosporins fluoroquinolones) amongst the uropathogenic *E. coli* isolates that varied from 76.51 to 100 (Annapurna et al., 2014; Niranjan and Malini, 2014; Ranjini et al., 2015) that was very similar to our study which cause difficulty in clinical management especially in a resource poor country, India.

It is of particular concern that resistance to β-lactams and fluoroquinolones is frequently driven by plasmid-borne extended spectrum β-lacatmase genes and plasmid mediated quinolone resistance (PMQR) genes in this uropathogen (Tayebi et al., 2016). Various reports suggested acquisition of plasmids of varied size and numbers associated with multiple drug resistant genes among pathogenic *E. coli* worldwide and nationwide (Sharma et al., 2010; Khadgi et al., 2013) with incidence of a plasmid band at 26 or 21 kb, respectively (Jan et al., 2009; Khadgi et al., 2013). In this study the number and size of the clinical plasmids varied with predominance of a ∼12 kb band. Incidence of two or more plasmid borne β-lactamase (*bla*_TEM_, *bla*_CTX–M_, *bla*_OXA_) and either one or two PMQR (*aac-1b-cr*, *oqxAB*, *qnrB*, *qnrS*) genes was observed. The resistant genes were successfully transmitted by conjugation in an environment irrespective of ceftazidme/ciprofloxacin selection. Successive co-existence and co-transmission (Dhawde et al., 2018) of these resistant determinants were previously reported that poses difficulty in health care management. Random administration of the respective group of drugs facilitates mobilization of plasmids that carry resistance genes by horizontal gene transfer (HGT) through natural mechanisms such as conjugation or transformation (Davies and Davies, 2010). Additionally resistant plasmids confer positively selectable phenotypes with the presence of antimicrobial resistance genes. Other essential regions of plasmid include their replication origin involved in replication and genes that are involved in the formation of *trans*-envelope machinery and pili structure that includes Type IV secretion system (T4SS), a large macromolecular machinery which control conjugation and dissemination of antibiotic resistance (Fillox, 2010; Cabezón et al., 2015). T4SSs are implicated not only in bacterial conjugation but also in the secretion of virulence factors to the host. IncF, IncP, and IncI conjugation systems were reported in *E. coli* that were further related to plasmid incompatibility groups (Hazes and Frost, 2008) which were ascertained to understand resistance plasmid epidemiology, as well as strain epidemiology (Edwards and Holt, 2013). Additionally the T4SSs encoded in plasmids with IncI conjugation system have a number of physical and functional features that distinctly distinguish them from the IncF and IncP systems in the *Enterobacteriaceae* strains (Hazes and Frost, 2008).

In this study predominance of multiple replicon types were observed among individual plasmid screened from the transconjugants irrespective of ceftazidime/ciprofloxacin selection although the number increased in the latter. Acquisition of resistant plasmid with multiple replicon types were reported (Huang et al., 2012) that may be attributed to frequent recombination events under antibiotic selection which creates difficulty in typing the resistant plasmids and their mode of dissemination. The random recombination events may also result in re-shuffling of resistant genes under antibiotic pressure during selection of transconjugants. However, conjugative-plasmid transfer in the gram-negative bacteria was observed to be related to contacts created by the conjugative pilus machinery which was constitutively expressed and independent of antibiotic selection (Lopatkin et al., 2016). Pili and pili associated gene types assisting in conjugal transfer of resistant plasmids were thought to be universally present amongst these plasmids that disseminated by IncF, IncP, and IncI conjugation system, respectively. A variety of mechanisms and structures involved in the transport of DNA amongst bacteria was dependent on the assembly of a pilus, be it an F, P, I, or T pilus, which brings cells together and provides an interface to exchange macromolecules directly from cell to cell (Hazes and Frost, 2008; Shintani et al., 2015).

Nucleotide sequences of genes encoding pili and pili associated genes identified from annotated plasmids R100, R64, R46, R388, R27, RA1, R6K assigned to IncF, IncI, IncN, IncW IncH, IncA/C, and IncX plasmid replicon types, respectively (Burmølle et al., 2012; Carattoli et al., 2014) revealed patterns of association that corresponds to those of the constituent single family replicons like IncF types (IncFrep, IncF1B, IncF11S), IncI, IncN, IncW, IncH, IncA/C, IncX associated with *traF* (Arutyunov et al., 2010), *pilM* (Kim and Komano, 1997), *traE* (Dolejska et al., 2013), *trwJ* (Liosa et al., 1991; Yeo et al., 2003; Revilla et al., 2008), *trhA* (Rooker et al., 1999; Sherburne et al., 2000), *traG* (Fricke et al., 2009), and *pilX4* (Burmølle et al., 2012) genes related to the conjugation system types respectively. In this study absence of *traE*, *trwJ*, *trhA*, *traG*, *pilx4* and presence of *traF* and *pilM* types among the resistant transmissible plasmids selected individually against ceftazidime and ciprofloxacin from all 19 transconjugants indicated that either IncF or IncI conjugation system was involved in the dissemination of the resistant determinants respectively. Conjugation system followed during the transfer of individual plasmid with multiple replicon type to the recipient strain was identical (F-type) in 13 out of 19 of the donor strains irrespective of the drug selection pressure. Moreover variation in the type of conjugation system (IncF/IncI) with respect to drug selection was observed in six clinical donors during conjugal transfer of genetic material to the recipient strain. Hence our study indicated that plasmid isolated from MDR uropathogenic *E. coli* obtained from hospitalized patients from the eastern part of India showed successful transmissibility by IncF and IncI conjugation system, the former being predominant. As bacteria have developed sophisticated ways to select, attach and infect their target cells, so adhesive and secretion pili serves as key protagonists during these events (Craig et al., 2004; Burrows, 2005; Hazes and Frost, 2008). Therefore pili and pili associated genes in transmissible resistance gene-carrying plasmids irrespective of their incompatibility types drives the success of recipient strains toward dissemination of drug resistance. Furthermore the results of this study also indicated that the type of conjugation system was independent of the associated resistance genes and the drugs used for selection that implied acquisition of resistance genes to be random, however, their dissemination was dependent upon the pili type related to T4SS of the transmissible plasmid that harbored the resistant determinants. Therefore the strength of this study relies on introduction of a novel technique of bio-typing plasmids of multiple replicon types in uropathogenic *E. coli* isolated from clinical settings. However, there were limitations as this study was conducted on a small pool of uropathogenic *E. coli* isolates. Moreover the conjugation system of plasmids without Inc group (non-typeable) could not be addressed by this technique due to their absence in the limited pool of isolates investigated.

## Conclusion

In summary, this is the first study of its kind which addressed novel technique of bio-typing plasmids of multiple replicon types in uropathogenic *E. coli* based on pili and pili associated genes. These plasmids were multidrug resistant and transmissible by conjugation, although the dissemination was independent of the associated resistance genes.

## Ethics Statement

Ethical approval was obtained from the Institutional Ethical Committee of the Calcutta School of Tropical Medicine (Kolkata, India) constituted under Order No. 1006 dated May 23, 2009. According to the standard guidelines of the ethical committee, the participants were duly informed and a written consent was obtained from them.

## Author Contributions

MM and SM conceived and designed the experiments, wrote the manuscript, analyzed the data, and read and approved the final manuscript. SM performed the experiments.

## Conflict of Interest

The authors declare that the research was conducted in the absence of any commercial or financial relationships that could be construed as a potential conflict of interest.

## Footnotes

1 http://blast.ncbi.nlm.nih.gov/

2 https://www.ncbi.nlm.nih.gov/nucleotide/

3 http://www.ebi.ac.uk/Tools/msa/clustalw2/

4 http://primerdigital.com/fastpcr.html
