# Supplementary material for: Characterization and Bio-Typing of Multidrug Resistance Plasmids From Uropathogenic Escherichia coli Isolated From Clinical Setting
Source: Front Microbiol. 2019 Dec 18;10:2913. doi: 10.3389/fmicb.2019.02913 (PMC6930805; doi:10.3389/fmicb.2019.02913)
Supplement: Supplementary file 1 [file Data_Sheet_1.docx]

**SUPPLEMENTARYTABLE 1-**Primers used to determine the chromosomal genes

| Genes | Primer sequences (5’-3’) | Amplicon size (bp) |
| --- | --- | --- |
| *fimH* | F-TGCAGAACGGATAAGCCGTGG  R-GCAGTCACCTGCCCTCCGGTA | 508 |
| *papC* | F-GACGGCTGTACTGCAGGGTGTGGC  R-ATATCCTTTCTGCAGGGATGCAATA | 328 |
| *cnf1* | F-AAGATGGAGTTTCCTATGCAG  R-TCAGAGTCCTGCCCTCATTAT | 498 |

**SUPPLEMENTARY FIGURE CAPTIONS**

**SUPPLEMENTARY FIGURE 1.**Agarose gel electrophoresis of multiplex PCR amplified product of chromosomal gene (*fimH*/*papC* ) using (A)genomic DNA as a template isolated from the clinical isolates used in the study and (B) plasmid DNA isolated from their respective transconjugants. Lanes1-19 represents the presence (A) and absence (B) of PCR amplified *fimH*(508bp) and *papC* (328bp) gene respectively. Lane M=100bpDNAladder.

**SUPPLEMENTARY FIGURE 2.**Agarose gel electrophoresis of PCR amplified product of chromosomal gene *cnf1* using (A) genomic DNA as a template isolated from the clinical isolates used in the study and (B) plasmid DNA isolated from their respective transconjugants. Lanes1-19 represents the presence (A) and absence (B) of PCR amplified *cnf1*(498bp) gene respectively. Lane M=100bpDNAladder.

**SUPPLEMENTARY FIGURE 3.** Restriction digestion pattern of genomicand pasmid DNAisolated from a transconjugant. Lane 1 of (A) and (B) represents uncut genomic DNA and plasmid DNA, Lanes 2-4represents restriction digestion of the genomic DNA and plasmid DNA for 6 hours at 37˚C by EcoR1, BamH1 and HindIII respectively.Lane M=λ/HindIII DNA ladder.

**SUPPLEMENTARY FIGURE 4**. Nucleotide sequences of genes, A; *traF*, B; *pilM*, C; *traE*, D; *trwJ*, E; *trhE*, F; *traG*, G; *pilx4* encoding pili and pili associated proteins from annotated plasmids R100, R64, R46, R388, R27, pRA1 and R6K respectively. Primer sequences are underlined using as forward primer (fp) and as reverse primer (rp).

**SUPPLEMENTARY FIGURE 1**.

A

1 2 3 4 5 6 7 8 9 M 10 11 12 13 14 15 16 M 17 18 19 M


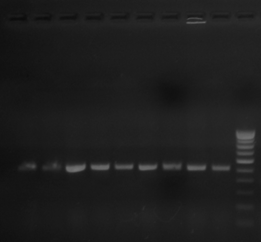

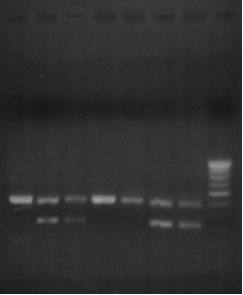

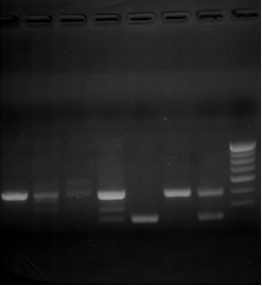


328bp

508bp

B

1 2 3 4 5 6 7 8 9 10 11 12 13 14 15 16 17 18 19 M


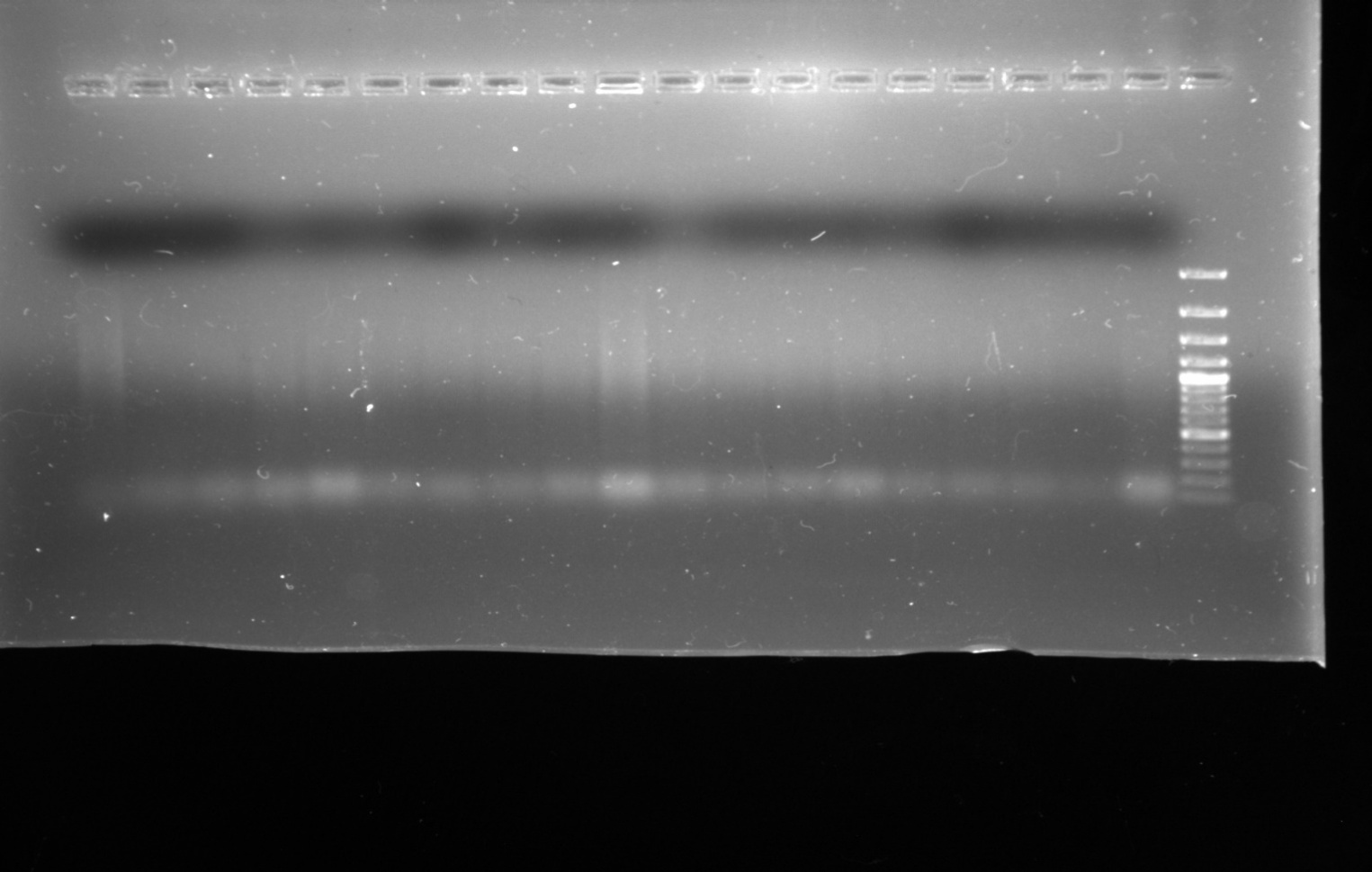


**SUPPLEMENTARY FIGURE 2**

A

1 2 3 4 5 6 7 8 9 10 11 M 12 13 14 15 16 17 18 19


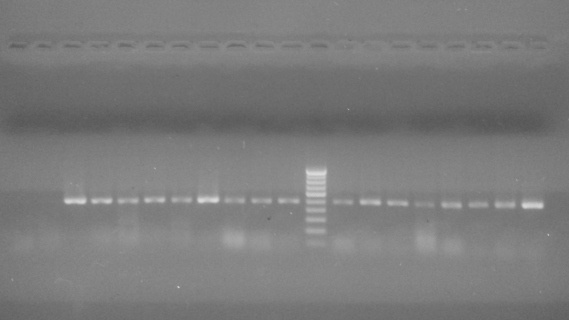


498bp

B

1 2 3 4 5 6 7 8 9 10 11 12 13 14 15 16 17 18 19 M


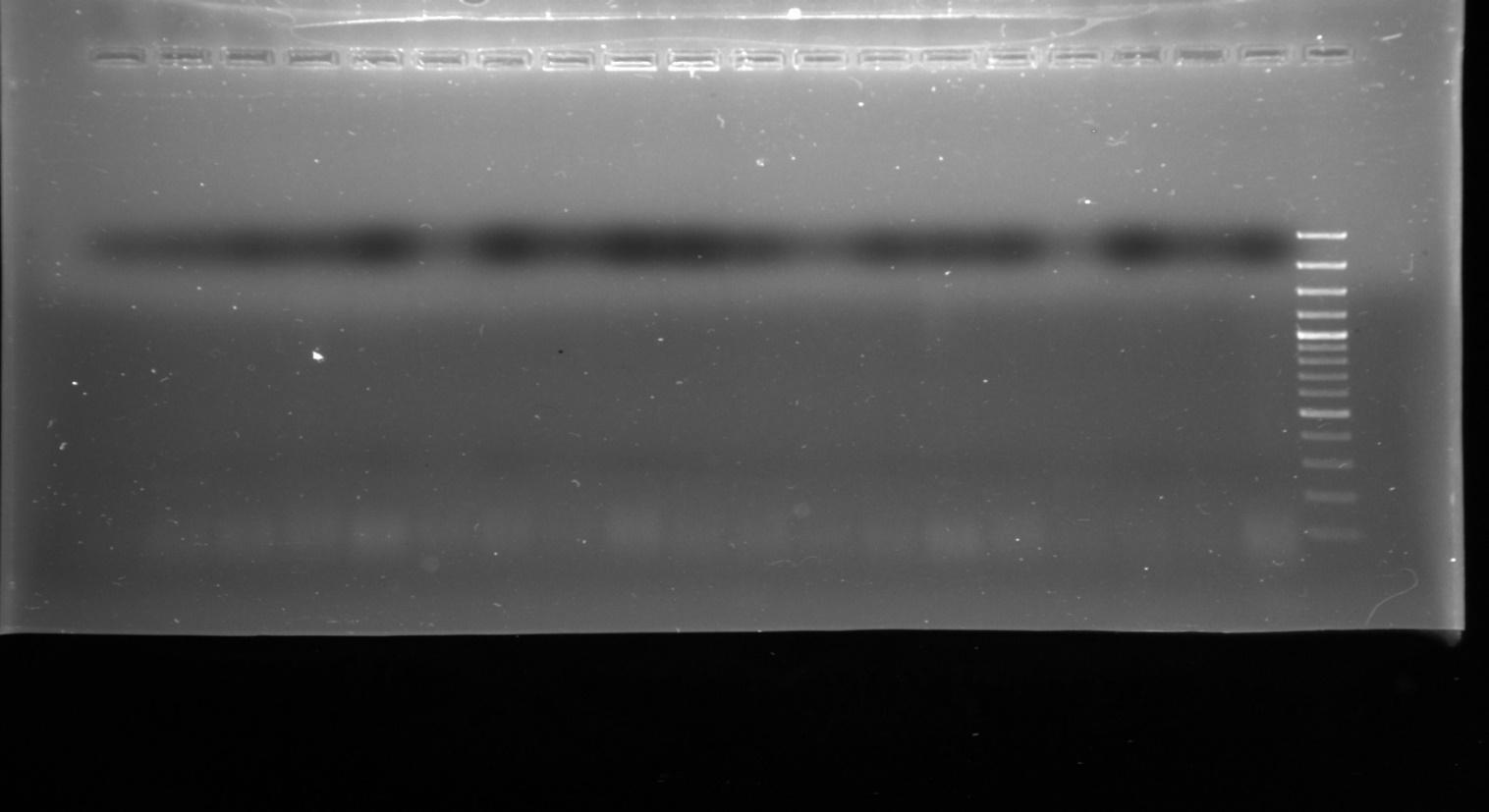


.

**SUPPLEMENTARY FIGURE 3**

M(Kb) 1 2 3 4

M (Kb) 1 2 3 4


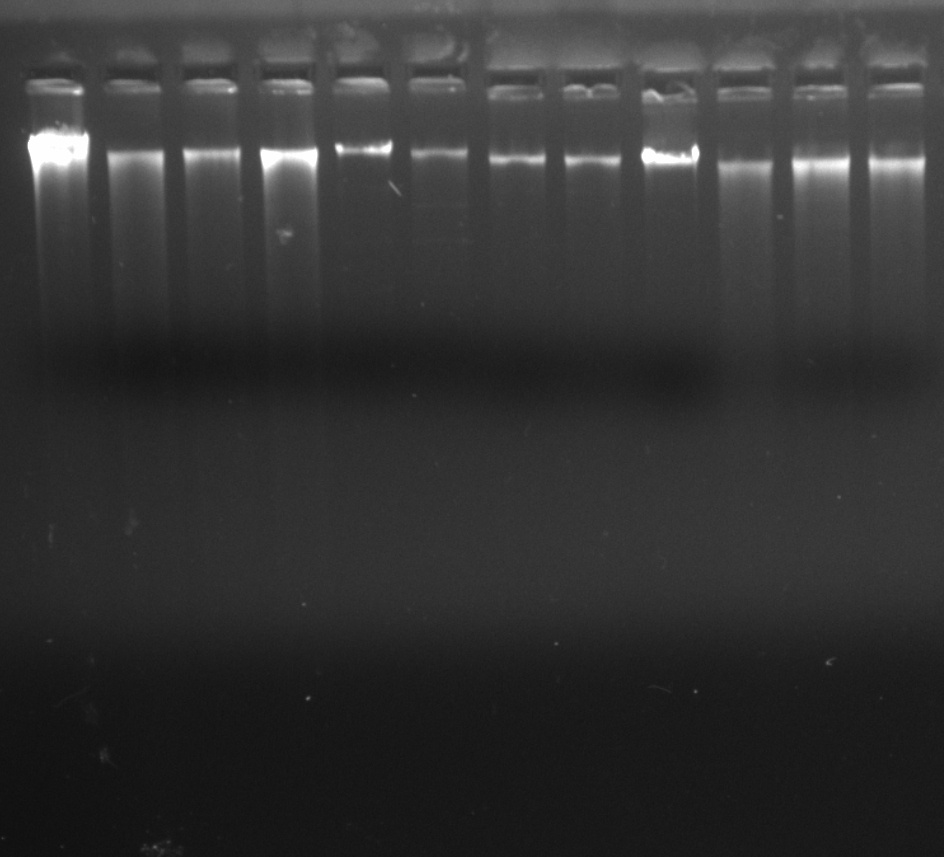

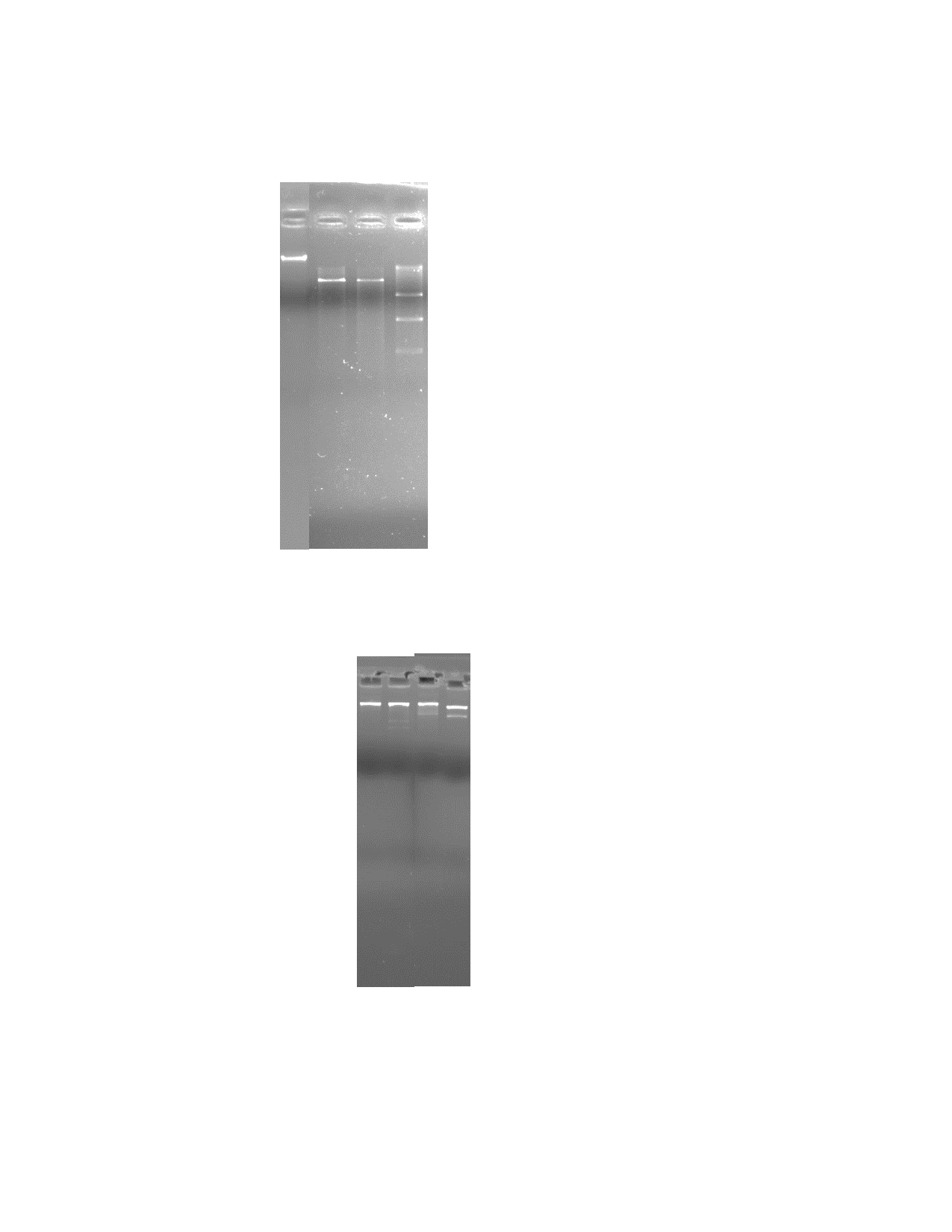


23.1

9.4

6.5

4.3

2.3

2.0

0.5

23.1

9.4

6.5

4.3

2.3

2.0

0.5

B

A

**SUPPLEMENTARY FIGURE 4**

A

>NC_002134.1 : (67095-67856) *Shigellaflexneri* 2b plasmid R100 DNA, complete sequence

**ATGATGAAAGGAATGAAGATGAATAAAGCATTACTGCCACTGTTACTCTGCTGCTTTATTTTTCCGGCGT**

**CAGGGAAAGATGCAGGCTGGCAGTGGTATAACGAGAAAATAAATCCGAAGGAAAAAGAAAATAAACCTGT**

**ACCTGCAGCCCCCCGTCAGGAGCCGGATATTATGCAGAAACTGGCCGCGCTGCAGACGGCAACAAAGCGG**

**GCGCTGTACGAAGCCATTCTGTATCCCGGCGTGGATAATTTTGTTAAATATTTCCGGCTGCAGAATTACT**

**GGACTCAGCAGGCCGGGCTTTTCACCATGAGTGCTAAAAAGGCCATGCTGGCACATCCCGAACTGGACTA**

**TAACCTGCAGTACAGCCATTACAACGGCACGGTACGGAACCAGCTGGCGGCAGACCAGGCGCAGCAGCGA**

**CAGGCCATTGCGAAACTGGCTGAACACTACGGCATCATGTTCTTTTACCGGGGGCAGGACCCCATCGACG**

**GGCAACTGGCGCAGGTCATTAATGGCTTCAGGGATACCTATGGTCTGAGTGTTATTCCCGTTTCCGTGGA**

**TGGCGTGATTAATCCGCTGTTGCCGGATTCCCGGACTGACCAGGGGCAGGCGCAGCGCCTCGGTGTGAAA**

**TATTTCCCGGCCATGATGCTGGTTGACCCGAAACAGGGCAGTGTTCGCCCGTTATCATACGGCTTTATTT**

**CACAGGACGACCTGGCAAAACAGTTCCTGAACGTTTCTGAAGATTTTAAACCGAATTTTTAA**

B

>AP005147.1:c114591-114154 *Salmonella enterica* subsp. entericaserovarTyphimurium plasmid R64 DNA, complete sequence

**ATGGGATGGCTGGTCATGGCCGTCGGCCTGACCATTCTGATTATCACTGGTAGCTACCAGAATCAGAAGA**

**TGTCTGAAACCACAAACGCCCAGCAATATGCAAGTGCGTCTGTATGGGCAAGTCAGATACTGATGATTGC**

**TAACAGAATCAATGATATCAGATACGTATCAGGCCAGCAGGACGGAGTAATTTCCTCAGATAAACTGGCT**

**CTCCCTGTCACTCCGGACTCCCGAATTAAACATCAGTTACAACAGGGGCGTCTGTGGGTATGGATGCCTG**

**AACAACCAGGTCTTGTTGAAACTTTGCGTTCAAAAAGTCGTGGTTCTGCACTAATAGGGATCTTCCAAAA**

**CGGGCAACTCACCTGGCTATCAGGAACTGCAACCGGTCTGACACCCCCGGCAGGCATTACTGCTGGTTCT**

**GTCGTCTACGTTAACTAA**

C

>NC_003292.1:9694-10392 *Salmonella enterica* subsp. EntericaserovarTyphimurium plasmid R46, complete sequence

**ATGAAAGCTAATAAAAAAACAGGGCTTACACGTGAAGCCATTAAAGAGTTCAACGAAAGCCGTAAAGGGC**

**TTGAAGTTGATCTGATGGATGAAGTGCTGAAGTCCCGGCGTACCGCCTGGATGGTTGCCACCGGTTCAGC**

**GGTGGTAACTGTTTTTGCACTCTCTTTAGTTGGTTACGTAGTGCATAAGTACAGCCAGCCAATCCCCGCA**

**CATCTGCTAACGCTCAACGAGGCCACTCACGAAGTACAGCAGGTCAAGCTGACCCGCGACCAGACCTCTT**

**ATGGTGACGAAATTGATAAGTTCTGGCTGACACAATATGTCATTCACCGTGAGAGCTATGACTTCTATTC**

**AGTTCAGGTCGACTATACGGCCGTTGCGTTAATGTCCACGCCGAACGTGGCAGAGTCTTACCAGAGCAAG**

**TTCAAGGGCCGCAATGGTCTTGATAAGGTTCTGGGCGACAGTGAAACAGCCCGCGTGAAGATTAACTCTG**

**TGATCCTCGATAAACCGCACGGCGTACGAACAATACGCTTTACTACGGTTCGCCGCGTGCGCACGAATCC**

**CGTTGATGATCAGCCGCAGCGCTGGATTGCCATTATGGGGTATGAATATAAATCGCTGGCGATGAATGCT**

**GAGCAGCGTTATGTCAACCCGCTGGGTTTCCGCGTGACGAGTTATCGCGTCAACCCGGAAGTTAACTGA**

D

>NC_028464.1: 5206-5886.*Escherichia coli* plasmid R388 DNA, complete sequence

**ATGAAGAAGCTGGTTATGACGGCGGCGGTGGCCGCCATTCTTGGGGCGGCAAGCCCTGTAATGGCCCAGG**

**GTATTCCTGTTTTCGACGGCACGCGGGCACTAGATTTTGTCCAACAGTTTGCCCGCATGAAAGAACAGCT**

**CGACACGGCCAAAGATCAGCTCGCCGAAGCGCAGCGCATGTATGAGGCCGTCACGGGTGGCCGTGGTCTT**

**GGGGATTTGATGCGCAATGCGCAACTCCGCGAATATCTGCCGGATGATTTGCGCACTGTCTATGACTCGG**

**CCAATGGTGGCGGGTATTCGGGCATTTCTGGCTCGATCAATGACATTCTGCGGGATGAGCGCCTTAACGG**

**CTCCGTGGCCGATATGCGCCGCTCTATCGAGGAACGTAGCCGCACGGCGCGGACAACCGACAAGGCCGTT**

**GGCTGCGGGCCTACGAAGGGGCACAGCAACGCCTCACGCAGATTGAAGGATGAAATTAGCCGTACCCAAG**

**ACCAAAAGGCCATTGAAGAATTGCAAGCCCGTATCGCGGGCGAGCAAGCCGCTATCCAGAACGAAACGAC**

**GAAGCTGCAAATGATCGCGCAACTGCGGCAAGCAGAACAGGCGTTGATTTCGGAGCAACGACGCGAGCGC**

**AACATGCCGATCCTGAGTAGTGGAAACCAAGGAATGCCGACTATCCAATGA**

E

>NC_002305.1: c38038-37685 *Salmonella typhi* plasmid R27, complete sequence

**ATGAAACTTCTCAGCAGGTTAAAAATTCCATTCATTAAAAATTCCAGTAATGGCGATTTTGATAACAAGG**

**ATGAGACTTCGCAAAAAGGAGGTGAAGAAGTAAAAGGAAGTTTTCTGGATTCAAAAGCACGCTTTAGTAA**

**AGAAATTGAAGGTTCTGAAATTGGAATTACATATAGTGCATTGATTAAACGCGACGAAAAACTCCTACGT**

**GTGAATACAGTTGCAATGATCATTATTGGGGTTCTGGTTGTAAAAAATCAATTTCTCACTGATCCTGTGA**

**CTATAGTGCTACCGCCAAATATGACGGAAGAAGTCAAAGTTGTTGGAAATAAAGCCTCAGAATCATATAA**

**AACGCAGTGGGCCTTATTCTTTAGTACTTTGATTGGAAACATAAACCCAACAAACATCGGTTTTGTTACA**

**ACAACTATATTGGATGCGCTTTCTCCGGATTTACAGGCAAAGACCCGGGAATCACTACAGCAGCAAACCA**

**ATATAATGCAGGCTCGTGGTGTAGAGCAGTCATTTAAGCCGATCGATATGTATTACGACACCAAAAATGA**

**CATGGTCTATGTCTGGGGAACAAAGTCAACTCGTTTGATTAATGTTCCGGACAAAACTGAATCATCCAAA**

**TGGACGTTTGAATGGGTCCTGGGAATGAAAAATGGACGACCGCGCATAGCCTATGTGAACCAGTATTCAG**

**GTACACCAAATATTAAGAAAATTACAATCAACGGCAAAGAACAACTGGCAACACTCGATAATCCGCCGCC**

**GTCAATAGGTAACTAA**

F

>NC_012885.1: 133514-137119 *Aeromonashydrophila* plasmid pRA1, complete sequence

**ATGGGAGCTTTTTCAATCCACTCTATCGGTGACTCTGCTTTTCTGGAGCAAATCCTGATTGCAGTATCAA**

**TGATCACCGGCACCGGAGACTTCGAGAAGATGGTCAGTATTGGCCTGCTTCTTGGGATCTTGATGATCTG**

**TATCCAGTCCGTCTTTCAAGGCGCGAAGCAAATCAATATCCAGCAAGTGCTGGTAGGTTGGATCATATAT**

**GCCTGTTTCTTCGGCCCGACCACAACGGTGACTATCGAAGACGCTTACACAGGGCAGGTCCGAGTCGTCG**

**CCAATGTGCCTATTGGAGTAGGCTTTGCTGGTGGTGTCATATCTAACGTGGGATACACCATCACCAATTT**

**GTTTGAAACTGGATATGGTGTAATCGTACCCAATGTCACGGAAAGCCACTTCTCAGAAACACTGAAACTT**

**TTGAATGACGTTAGACGGCGGGCCTATGACACCGGGGTTTTCACTGCGCTTAACTCAGCAAATGGTGGCG**

**GCTATGTTGACGTGAGACGTTCCTGGAACAATTACATCCGGGAATGCACATTAACCAAAGTTGATCTCAA**

**CCTAATGTCCCTTGATGAGTTGATGAATCGTTCAACCGACACGGCTTTGCGGTTCAACTCTCAGCTCTAC**

**GGAACCCGGTTGTATTTGTCCACATCCAACCCTGACGGCACTGACTACACATGTACCGATGGATGGGTGG**

**CTATTAGCACAGCAACTGCCAACCTAAGCAGCCCGGTTGTTGTTGATGCTCTAAACAGCCTACTGGGTAT**

**TGACACATCAACCGGAGACAACGCTCTAACGAAGCTGACTGATTCGCTTCAAGCGATGGGTGCCACAACC**

**ACCTCATCAATCGACTACCTGAAAGCCGCCGTTCTTGAGCCTCTGTATTATGAGGCCGCAGCAGGACGTT**

**ATCAGGACCTCCAGGACTATGGCTCTGCATTGATGATCAACCAAGCCATTCAGCAACGGAACACGCAGTG**

**GGCCGCAGAACAGTCGATGTTCATGACCGTCGTCCGACCAATGCTGACATTCTTTGAGGGGTTCATTTAC**

**GCTATCACCCCGATCATTGCTTTTATTATCGTGATGGGCAGCTTCGGCATCCAGTTAGCCGGTAAATATG**

**TACAAACCATCCTCTGGATTCAGCTATGGATGCCAGTCCTCTCAATTATCAACCTGTTTGTTCATACCGC**

**CGCGTCAAATGAGATGTCTAGCCTCAGTGCTGGAGGTCTCAACTCCATGTACGCGCTTTCCTCAACTGGA**

**GATGTGCTGCAACACTGGATTGCAACCGGCGGCATGTTGGCTGCGGCCACCCCGGTCATTTCCCTGTTTA**

**TCGTTACAGGTAGCACTTATGCCTTCACCAGCTTGGCATCGAGAATAAGTGGTGCTGACCACGTTGACGA**

**AAAGATGCAGACGCCAGATCTACTCAAGCAAGGTCCGGTTATGCAAAGTCAGCCAGCATACAATCACAAC**

**CAGTTTAGTGGTGCGATTGCGAACGGCGCAGAAAGCATGATCAGCACCTTCTCGCTTGGCTCCACTCTGG**

**CATCAGGTGTGAGCTCCGCACAGGCATTACAAAGCCAGAAATCGGAGGCCTTCCAAAGCACCCTCGGTCG**

**AGGTTTTTCTGATGGAGTAAGTCAGGATCAAGCCTACTCAAGACTCTCCAATGTTGGGCGCAACGTTTCG**

**TCTCAAAGCACAGCTCAGAGCCAATTGATCAACCAACAAGCCAAGAACTTCATGGATAAGTTCCAAGTGG**

**ATGATAGCCACTCTGATGCAGTCAAAGGTGCTTTTGCCATGCAGGCTATGGGCACTCTCGATGCTGACGA**

**AGCGGCTTCCATGCTTATGCCTATGGTTGGTAAGGTCAGGGCGGCAATGAAGGGGGCTGCTGGTATTAAA**

**TCAAACAGTACAGCACTTGTTCCTACTGGTGGTGATGGCGAATCAGGCGATGTGCTGGACATCAAAGCGC**

**AAGCGAAGGGGGCAACAGAGTCCTCAGCTCAAGACTCCTCAAGCTGGTCAGCAAGCGATGTGTCTCAGTT**

**CATGAAGGGTGTGAGTTATTCACAAACCGATAGCCAAGCGTTGACTAATCAATTGGCACAGGGTTTTAGT**

**CGTTCCGGTAGCGAGTCATTTAAACAAACCTGGGGGGATAGCTTATCCCAGAACCTGTCCAAGTCGGCCT**

**CTGAACTGGTGTCAGCATCCGACACCTTCACAACAATGAGCCAACTCCAGAATCAAATGGGCTCCATGAC**

**TAACACCGACTTTAAAACTCTCGGTGGTGCAGTGGCACAAACCCCTGAGGCCATGAATCAACTGAATGAC**

**TATTTCCGAAATGCCGCTCCGCAGTCGGTTAAAGACGAGGCTGCTTCACTACAGCAAAGATACCAAGCCT**

**ACGGAATGTCGCCTCAGGTGGCCCAAGCAGCAGCCCGAATGACAGCAATGACCAACTCCAAAAACTATGA**

**TCCGGGTAAGGAGCTTGGCGGGTATCAAGCTGCACTACAGGCAATCAATACTGCGTCCGGGCGCAACGGA**

**TCCTTTAGTGGTGACGCCTACGGAAATAGCGGTATTGAAGGCCCGAATGTTCAAGGTCTACCAGGTCAGG**

**TTCAAGGTGCTGTAGGCAGTGGACCTAATATCCCAACCGGATTCAGAGAGAATGTGGCTGGGATGGCTGG**

**AACTAATCCGGCATCCGAAGCAGGCCAGTTACCAACGAACAGCCCCCTTGTTCAAAATGAACATGCAGCA**

**GGTACATCAGCTCTTCACAACCTGGCGCAGCAAACAGAGCGAAACGTAACTGCTCCCGAACTCAAAAAAG**

**CACAAGATAACCTCATTAACTCGCTTCCGGAGATGTCTTGGAGCGCTTCGGCATGGGGGGCGTGGGATAA**

**CTTCAGTGACAAGATGGGCCGCAGAGCTGAACAAGCAGGCGGGGCTCTCATTGCTGGTGGTCAGGCTGGC**

**GCTGATGCGTTCTCACAAGCAATGGATCAAATGAGAACGATGACGCCTGAGCAACGCGACCAATTCGTCG**

**CGGCAACTCAACGCGGCGATCAGGCAGTCAAAGAAGAGTTTGGCTGGGCCGGGGGCGCGATGGTTGGAAT**

**GGCTAAACTTGGCCGCAACGTCATGGGTGCTGCTGCAAGCGGCTATAGTGCAGCCAAAGAATGGTTAAGT**

**GGTAAATCAGACCTATCAGAAGCCGCTAAAGGGATGAGCATGGAGGAACGCGGTGCGTTCTATGCAGCAG**

**CGCTATCCTCTGCCGCAGAAGCCGGTGGTGGAGCAGCACAACAGTTCATGAACCAGTATGGCGATGAGTT**

**CAAAGAGACAATGCAGTCAATTGCTCAAAACCGCTATGGACTTACTCAAGGGCAAGCTGCTGTTTATGCT**

**GAGTCTTTTGATACAAACGAAGGCCGCATGAACCAGGCAATTCAGAATCTGAAAATGGAATATGCAGAAC**

**GTAACCCGGATGGTTCACCGATGACGCAAGGTGGTCAGCCTGTTCTTTCTCAGCAAAACGAAGAGTTCGC**

**TGACAAGCTGGTAAACGTATTGCAGAACTCGACGGGAGCTGGAGACCGTTCGGGGAGTTATTTGACTGCT**

**GTCAGAGGGTACAACATAGCAAATCAAAGGTTCTAA**

G

>AJ006342.1:2332-4779 *Escherichia coli*IncX plasmid R6K synthesis operon

**ATGAAGCTGAAAGACCAGGCGTCTATTGAAGCTCTTATCCCTTACTCTTCTCACATTACTGATGATTTAG**

**TTGTCACCAAAAACCGCGATTTGCTCGCAACGTGGCAAGTTGATGGTGCTTATTTTGAATGTGTGGATGA**

**CGAAGATTTAACGTTACTTACTGACCAGTTAAATACGCTCATTCGTAGTTTCGAAGGCAGGGCGATAACG**

**TTCTATACTCACCGCGTGAGGGTAAGAAAAGAAGTTAAGGTTGGTTTTAATAGCAAGATTCCTTTCGTTA**

**ATCGCGTAATGAATGATTACTATTCCTCCCTTTCTGAGCCTGAGTATTTTGAAAACAGACTCTATTTAAC**

**GATTTGTTATAAGCCTTTTAATATCGAGGATAAGGTTTCTCATTTTATCTCGAAGAATAAAAAGGAAAAT**

**AATATCTTTGATGAGCCAATCAATGATATGAATGAAATTTGCGGGCGACTTGATACATATTTATCTCGTT**

**TCCATGCACATCGTCTTGGACTGGTTGAGGAAAACGGGCGAGTTTTCTCAGATCAGCTATCCCTTTTTCA**

**GTATTTGCTTTCTGGTAAGTGGCAGAAGGTCAGAGTTACAAATAGCCCTTTTTACACTTATCTGGGGGGC**

**AAAGATTTGTTCTTTGGAAATGATGCCGGACAGATTACGGCGTCGCAGAATGCCCGGTATTTCCGTTCGA**

**TAGAAATCAAAGATTACTTTCAGGAAACAGATGCAGGCATTTTTGATGCGCTCATGTATCTTCCTGTTGA**

**GTATGTCTTTACATCGTCTTTCACGTCGATGGATAAGCAAGCCGCTGTTAAAGCGCTTGATGATCAGATA**

**GATAAGCTTGAACTGACGGATGACGCTGCTAAATCCCTCCTTGCTGATTTGCGCGTTGGTCTTGATATGG**

**TTTCAAGCGGATACAACTCTTTCGGAAAGTGTCACCAAACATTGATTGTCTTTGCTGACACGCCGGAACG**

**CTTGGTGAAAGATACGAATATCGTTACTACGACCTTAGAAGACCTGGGGCTGATTGTTACTTATTCTACC**

**CTAAGCTTGGGTGCTGCCTTTTTCTCTCAGTTGCCAGGCAATTACAATCTTCGTCCTCGCCTAAGTATGC**

**TGAGTAGCCTCAACTTTGCCGAGATGGAAAGTTTCCATAACTTCTTTCACGGTAAAGCTACTGGTAATAC**

**TTGGGGCAATAGCTTAATGGCTCTTCGCGGCTCTGGTAACGATGTCTACCATCTCAATTATCACATGACT**

**ACGGAGAATATTAACTTTTTCGGCAAGAACCCTACGCTTGGCCATTGTGAGATCCTGGGAACCTCTAACG**

**TAGGTAAAACCGTTCTCATGATGATCATGTCTTATGCGGCACAGCAATTTGGTACGCCTGAATCATTTCC**

**GGAAAACCGAAAAGTAAGGAAGCTAACCACAGTCTTCTTTGATAAGGACAGAGCGGGAGAAGTGGGTATT**

**CGTGCTATGGGCGGGGCTTATTTTCGTGTGAAGGGCGGAGAGCCTACCGGGTGGAACCCGGCAGCCCTCC**

**CGCCAACTAAGCGAAACATAGCATTTGTAAAAGACATAATAAGGCTGATATGTAAGCTCAACGGCAATAC**

**GGTTGATGATTACCAACATTCGCTGATTTCTTCAGCCGTTGACCGTCTTATGCAAAGGGAAGACCGTTCC**

**TTTCCTATCAGCAAGCTCATCCCGCTGATCATGGAGCCGGATGATACTGAAACTAAGCGACATGGGCTTA**

**AAGCTCGACTGAGAGCGTGGAAGCAGGGGGGCGAGTATGGCTGGTTGCTCGATAATGCTTCCGATTCCTT**

**TGATGTTGCGCATTTGGATGTTTTTGGCATCGATGGAACAGAATTCCTTGATGATAAGGTTGTAGCCCCG**

**GTTGCTTCTTTTTACCTGATTTACCGGGTAACAATGCTGGCTGACGGTCGCCGTTTGCTGATCTACATGG**

**ATGAGTTCTGGCAATGGATAAACAACGATGCCTTCAAGGATTTCGTTTATAACAAGCTTAAAACTGGCCG**

**TAAGCTGGATATGGTGCTTGTTCCCGCAACGCAATCACCGGATGAATTAATTAAATCTCCAATTGCCGCA**

**GCCGTCAGGGAACAGTGCGCCACCCATATCTACCTCGCTAACCCCAAAGCGAAACGCAATGAATATGTTG**

**ATGAACTTCAGGTAAGGGATCTTTATTTTGACAAAATCAAAGCTATCGACCCCTTGTCTCGCCAGTTTCT**

**GGTAGTCAAAAACCCGCAACGGCAGGGCGAACGAGATGACTTTGCGGCTTTCGCAAAGTTAGACCTGGGC**

**AAAGCTGCCTACTACCTTCCCATTCTGAGTGCTTCAGCAGAACAACTTGAGTTGTTCGACGAAATCTGGT**

**CTGAAGGTATGGCACCTGAAGAGTGGATAGATACATATCTTGAACGGGCTAACCGTGGAGTCAAATAA**
